# “If I can accept my queerness, I can accept my body as it is”: Understanding weight-related perspectives and stigma from sexual minority women

**DOI:** 10.3389/fpsyt.2025.1687680

**Published:** 2025-10-02

**Authors:** Lauren A. Fowler, Yilin Wang, Catherine Wall, Allyson Velkovich, Erin N. Harrop, Melissa M. Vázquez, Janis Mensah, Annesa Flentje, Emily S. Mann

**Affiliations:** ^1^ Department of Health Promotion, Education, and Behavior, University of South Carolina, Columbia, SC, United States; ^2^ Brown School of Social Work, School of Public Health, Washington University in St. Louis, St. Louis, MO, United States; ^3^ Department of Psychology, Virginia Commonwealth University, Richmond, VA, United States; ^4^ Graduate School of Social Work, University of Denver, Denver, CO, United States; ^5^ Department of Psychiatry, Washington University School of Medicine, St. Louis, MO, United States; ^6^ Stanford Prevention Research Center, Department of Medicine, School of Medicine, Stanford University, Stanford, CA, United States; ^7^ Department of Women’s and Gender Studies, McCausland College of Arts and Sciences, University of South Carolina, Columbia, SC, United States

**Keywords:** weight stigma, sexual minority women, disordered eating, body norms, minority stress, structural stigma

## Abstract

**Introduction:**

Sociocultural norms that conflate thinness with health and morality contribute to widespread weight stigma, with well-documented consequences for physical and mental health. Sexual minority women (SMW), particularly those living in larger bodies, may be especially affected due to the intersection of heterosexism, sexism, and weight stigma across their lives.

**Methods:**

This qualitative study utilized life history-informed semi-structured interviews with 24 cisgender SMW, ages 22–46, to explore how they experience sociocultural messages about weight, body size, and health over time, and how these experiences intersect with other aspects of structural marginalization. Interviews were audio recorded and transcripts were coded using a reflexive thematic analysis approach.

**Results:**

Three overarching contexts were identified in which weight stigma is reinforced and resisted (1): dominant cultural norms—across media, healthcare, and public spaces—that moralized weight and pathologized larger bodies (2); families of origin, where intergenerational dieting, food restriction, and body surveillance reinforced weight bias beginning in childhood; and (3) queer communities, which sometimes fostered acceptance but also reproduced exclusionary body norms shaped by gender presentation, race, and size. Across settings, participants described the cumulative and compounding effects of stigma on mental health, including disordered eating. Their experiences also highlighted the complex role of sexual identity and queer community in shaping body-related experiences, which were affirming, marginalizing, and both simultaneously.

**Discussion:**

Our findings underscore the importance of applying intersectional and life-course frameworks and call for systemic changes in public health to shift from weight-centric approaches toward affirming, weight-inclusive paradigms that address interlocking systems of oppression.

## Introduction

Sociocultural ideals regarding body size, weight management, and “health” play a significant role in shaping our health behaviors, mental health, and overall wellbeing. Western social norms center body size as a reliable metric of health, equate thinness with moral virtue, and place responsibility for health as a moral imperative that individuals are responsible to achieve (1). These dominant narratives are reinforced through weight discourses disseminated across medicine, public health, and media platforms, contributing to a broader culture of diet and body surveillance (2). Individuals whose bodies are not thin, gender-conforming, and able-bodied, especially when they are non-white, often face body- and weight-related stigma. This stigma, manifesting as both interpersonal and structural prejudice and discrimination against people with higher weight (3) can be internalized, resulting in self-blame and self-hatred (4, 5).

Body Mass Index (BMI), a ratio of weight (kg) divided by height (m) squared and informed by observations of an astronomer and statistician to describe an “average” (white) individual (6), is a strong predictor of weight stigma, such that individuals who have higher BMI are more likely to face discrimination based on their body size or shape (7, 8). Weight stigma itself (and not higher weight) is associated with poor self-rated health, morbidity, and mortality. Other consequences of weight stigma include dysregulation of physiological systems due to chronic stress exposure (9, 10), internalized weight bias, and maladaptive weight-control behaviors, as well as cycles of repeated weight loss and gain that elevate health risks regardless of BMI (10). Finally, weight stigma also limits access to and utilization of healthcare services, yielding worse long-term outcomes (4).

Weight stigma experiences do not occur in isolation. Individuals who are multiply minoritized by race, gender, sexual orientation, social class, and/or ability may experience distinct forms of stress due to structural oppression (e.g., racism, sexism, heterosexism, classism, and/or ableism) that compound the effects of weight stigma and its consequences (11, 12). Intersectionality theory offers a valuable framework for examining how multiple systems of oppression interact to create unique and compounded experiences of harm at the intersections of race, gender, sexual orientation, class, and body size (13–15). In the context of weight stigma, this framework highlights how marginalized groups may encounter overlapping forms of discrimination that cannot be fully understood through single-axis analyses (16).

Focusing on groups who are more likely to experience both weight stigma and other forms of oppression provides greater insight into the diversity of ways individuals experience weight stigma and interpret sociocultural messages that can lead to health disparities. Sexual minority women (SMW—i.e., women who identify as a sexual orientation other than heterosexual, such as lesbian, gay, or bisexual) are more likely to have higher BMIs (17, 18), placing them at increased risk for experiencing weight-related stigma and its consequences. Moreover, SMW are disproportionately impacted by mental health disparities, including higher rates of depression and self-harm, disordered eating, and unhealthy weight-control behaviors when compared to heterosexual women; researchers attribute these disparities in part to minority stress (19). Sexual minority health researchers highlight how experiences of minority stress due to increased exposure to stigma and discrimination at the structural, interpersonal, and individual levels can place sexual minorities at greater risk for mental health disparities (20, 21). Structural stigma, or the “societal-level conditions, cultural norms, and institutional policies that constrain the opportunities, resources, and wellbeing of the stigmatized”, is especially relevant for understanding how weight stigma intersects with other experiences of oppression to shape SMW’s mental health (22).

While most research using minority stress theory has focused on sexual identity-related stigma, SMW also face intersectional stigma that shapes how they experience and respond to body and weight-related stigma. Weight stigma itself has been consistently linked to elevated psychological distress, depression, and disordered eating (23), yet little research has examined how these effects may be amplified when experienced in concert with other oppressive conditions related to social class, race/ethnicity, and/or disability. Intersectional approaches suggest that SMW’s experiences of weight stigma cannot be fully understood without considering how minority stress, including sexuality-related stigma, and weight stigma interact in unique, and potentially mutually reinforcing ways. For example, emerging evidence indicates that SMW may find the increasing medicalization of body size—whereby larger body size is framed primarily as a medical problem requiring intervention to treat (24)—less relevant (and potentially harmful) to their wellbeing (25).

Despite the emergence of critical inquiry in the social and health sciences examining the consequences of weight stigma among diverse women, this scholarship primarily relies on quantitative methods, overlooks intersectional frameworks, and underemphasizes structural-level contributors to health inequities for individuals living in larger bodies. Recent studies also suggest that racially minoritized women may experience different stigma processes compared to white women due to discrimination, socioeconomic disparities, and access to care (11, 19). Less attention has been paid to SMW, who are more likely to live in larger bodies than their heterosexual counterparts and are more likely to experience intersectional stigma and associated inequities. Centering SMW in research examining the relationship between weight stigma and mental health, with attention to interlocking and mutually reinforcing systems of oppression, can enhance our understanding of how stigma drives health disparities, and identify modifiable social-structural factors to promote health equity. This study contributes to new knowledge by using a life-course perspective and exploring weight stigma as the driver of adverse health outcomes as opposed to weight.

## Methods

### Study design and approach

This study used intersectionality theory, minority stress theory, and a life course perspective as foundational frameworks to understand the complexities surrounding weight stigma among SMW. We employed an epistemological approach that avoided moralizing questions and used non-judgmental language to increase comfort when sharing experiences of weight stigma. This approach centered participants’ lived realities, prioritized their perspectives over predetermined frameworks (e.g., weight-centric approaches), and sought to facilitate open, respectful explorations of weight-related experiences within the context of intersecting systems of oppression.

We aimed to examine how SMW perceived and experienced sociocultural norms and expectations around weight, body size, and weight management during their lifetime, with attention to intersecting structures of oppression related to gender, sexual orientation, and other social categories, including how body size-related discrimination is experienced. The first author conducted semi-structured life history interviews (approximately 60–90 minutes in length) with adult cisgender women who identify with non-heterosexual sexual orientations. Interviews examined how participants perceived the mental health impact of weight stigma and its intersections with other forms of discrimination. A Community Advisory Board, comprised of experts on queer health or weight stigma, and/or self-identified community members, were recruited through social media and word of mouth for the purposes of this project (*n* = 6). Council members provided iterative feedback on the research questions, eligibility criteria, and the semi-structured interview guide through independent review, team meetings, and individual meetings. All members were compensated for their time.

### Inclusion and exclusion criteria

Participants were eligible if they met the following inclusion criteria: 1) adults, aged 18 years or older; 2) identified as non-heterosexual (e.g., lesbian, bisexual, pansexual, queer, questioning, fluid, or report other non-heterosexual identities); 3) identified as cisgender women, and 4) were able to speak English. No additional restrictions were placed on the sample, including by BMI or body size. Exclusion criteria were: 1) under 18 years old; 2) exclusively heterosexual; 3) not cisgender woman; 4) could not speak English comfortably; and 5) not a current U.S. resident.

### Sampling strategy and participants

Twenty-four participants were recruited. To ensure a diverse sample, purposive sampling using a maximum variation strategy (26) was employed, prioritizing individuals with diverse sexual orientations, ages, weight statuses, and racial/ethnic backgrounds.

### Recruitment, consent, and study procedures

A study flier with a link to the screening survey was distributed through social media (e.g., Facebook, Instagram). Interested individuals completed an online consent form for both the screener and interview before filling out the screener, which included demographic information. Consent to audio and visual recording via Zoom was included in the informed consent. All procedures were approved by the IRB of record. All interviews were conducted virtually via video conference by the first author in a private setting.

### Interview guide

The semi-structured interview (see Appendix A for interview guide) was guided by a life history calendar approach (27), which is appropriate for research involving populations who are historically marginalized and/or have experienced trauma, including sexual minorities (28). This approach encouraged participants to identify salient life experiences and prompted participants to consider important moments in their sexual identity development and weight-related experiences over the life course. The life history approach is particularly appropriate for this study given that normative messages around health, appearance, and body image are influenced by early life experiences (29) and are related to chronic disease risk and disordered eating cross-sectionally and prospectively (30, 31). The preliminary interview guide (including questions and follow up probes) was developed from the extant literature on minority stress, weight stigma, body image development, and disordered eating behaviors, and existing instruments used in previous research, with a focus on identifying and probing for salient systems of oppression related to weight, race/ethnicity, gender, ability, and class that may intersect with sexuality. Community board members provided feedback on the preliminary interview guide and research questions. Following, we piloted this revised interview guide with three pilot interviews to clarify language and questions, and ensure the guide was of appropriate length for a 60-to-90-minute interview.

### Transcription, validation, and deidentification

Transcription type: verbatim with lexical repetitions preserved; non-lexical fillers (um, uh, coughs, sighs) omitted. Prior to analysis, transcripts were de-identified in accordance with standard ethical and privacy guidelines (e.g., replacing direct identifiers with coded labels, substituting personal names with descriptors (e.g., “participant’s friend”), and generalizing third-party names and locations. All de-identification steps were validated by independent review and by cross-referencing a subset of transcripts with the corresponding audio to ensure fidelity and absence of residual identifiers. Transcripts were de-identified and validated (checked against audio recordings for accuracy) prior to analysis.

### Epistemological approach and theoretical frame

In this study, we employed a consensus coding approach grounded in interpretivist and narrative frameworks, which recognizes that “truth” is subjective and shaped by individual perspectives and social contexts. Rather than relying on inter-rater reliability, which assumes a singular “true code” for each transcript segment, our methodology emphasized collaborative discussions among the coding team to develop a shared understanding of participant narratives. As different coders analyzed the data, they engaged in discussions to resolve discrepancies in code interpretation, ensuring that the analysis reflected the diverse viewpoints inherent in the participants’ stories. Ultimately, the coding team organized emerging codes into coherent themes through group discussions, which highlighted both similarities and differences in the participants’ experiences. This approach underscores our commitment to an inclusive and nuanced exploration of the data, ensuring that our findings are reflective of the complexities of the participants’ lived experiences.

### Thematic data analysis

Thematic analysis employed a constructivist-reflexive approach (32) facilitated by Dedoose software (33), blending inductive emergence with structured taxonomic development through three iterative phases involving four trained coders (L.A.F., Y.W., C.W., A.V.). Beginning with independent review of eight initial transcripts, the team generated ~100 preliminary codes encompassing descriptive categorizations, participant-derived *in vivo* language, and processual codes tracking stigma trajectories. Weekly analytic dialogues then organized these into a three-tiered taxonomy: externalized stigma (societal/interpersonal bias), internalized stigma (self-directed negativity), and *a priori* categories (protective factors, intersectionality), with non-stigma codes retained exclusively when contextualizing central phenomena such as mental/physical health comorbidities. The process culminated in intersectional mapping through six *a priori* domains selected for research relevance and data prevalence, narrative tracing of stigma experiences across life trajectories, and refinement cycles of dynamic codebook evolution. This iterative consensus-building preserved experiential diversity through constant comparison of linguistic patterns and situational contexts. The resultant hybrid methodology balanced inductive sensitivity to lived experience with deductive validation of theoretically significant constructs, particularly regarding intersectional stigma manifestations, with a consolidated codebook detailing operational definitions and decision rules available from the first author upon reasonable request.

The researchers identified key concepts that encapsulated participants’ experiences, such as “intersectional stigma,” “protective factors,” and “queer community.” These concepts were not merely emergent but were constructed through careful analysis and clustering of related codes. Codes that shared similar meanings or addressed common experiences were grouped together to form coherent themes. For example, codes related to classism, heterosexism, and ableism were clustered under the theme of “Intersectional Stigma.” The team considered candidate themes and checked the data to ensure they accurately represented the dataset and addressed the research questions. The research team revisited the transcripts to confirm that each theme was well-supported by multiple excerpts, ensuring that the themes reflected the diversity of experiences shared by participants. The refinement process involved iterating back to the dataset, modifying themes based on new insights, and ensuring that each theme maintained internal coherence while also reflecting the broader narrative of the participants’ experiences. Each theme was clearly defined and distinct from others, preventing overlaps. The themes were internally consistent, with all data extracts supporting the identified themes. Data extracts were used to illustrate themes, ensuring that the analysis remained closely tied to participants’ voices. Themes and selected illustrative quotes were organized into tables, with themes and corresponding quotes discussed in the text.

### Positionality and reflexivity

As a research team exploring intersectional stigma among the SMW community, we recognize that our perspectives are shaped by our lived experiences. The coding team was comprised of individuals who identify as larger bodied, big-sized, or mid-size/plus-size who have lived experience of weight stigma, and one member who is currently straight-sized. The coding team identifies as: heterosexual, heterosexual/questioning, queer, transgender, and cisgender, and team members were White and Asian, two with lived experience of disordered eating. No coding team members identify as disabled. The research team was comprised primarily of people who identify as a sexual and/or gender minority and non-disabled, and were predominately, but not exclusively, white and U.S.-born, with varied lived experiences of weight stigma and thin privilege. Throughout the coding and analysis process, the team regularly reflected on how our positionality impacted our perspectives (including areas of insight or biases), with special consideration of our experiences related to body-related oppression. This involved activities such as positionality worksheets, reflexive journaling memos, and regular coding discussions. In alignment with critical constructivist approaches, we explored and interrogated how our identities and experiences shaped our interpretations rather than assuming we could approach the data neutrally.

## Results

### Participant characteristics

Participants (*N* = 24) were cisgender women aged 22 - 46 (M = 32.0, SD = 6.3). Participants reported their race and ethnicity separately, with the option to select more than one racial category. Six participants (25.0%) identified as Hispanic/Latinx, (including multiracial combinations: Hispanic/Latinx only (n = 2, 8.3%), Hispanic/Latinx and White (n = 2, 8.3%), Hispanic/Latinx and Black/African American (n = 1, 4.2%), and Hispanic/Latinx, Native American/Indigenous, and White (n = 1, 4.2%). Non-Hispanic participants (n = 18, 75.0%), included White (n = 14, 58.3%), Asian American/Pacific Islander and White (n = 2, 8.3%), Black/African American and White (n = 1, 4.2%), and Native American/Indigenous (n = 1, 4.2%). Sexual orientation responses (multi-select allowed) included 12 pansexual participants (50%), 11 queer participants (45.8%), 10 asexual spectrum (ACE spectrum; e.g., graysexual, demisexual, asexual) participants (41.7%), eight lesbian participants (33.3%), six bisexual participants (25%), one biromantic (4.2%), and one same-gender-loving participant (4.2%). Most participants (58.3%, n = 14) had a bachelor’s degree or higher. BMI, calculated from self-reported height and weight, is presented here as a proxy for weight-based discrimination experiences, and ranged from 18.9 to 68.8, with a mean of 38.8 (*SD* = 13.6). See **Table 1** for sample characteristics.

**Table 1:** Sample characteristics (N = 24).

Characteristic	*N*	%	% of cases
Race^a^/Ethnicity			
Hispanic/Latinx			
Hispanic/Latinx	2	8.3%	
Hispanic/Latinx, White	2	8.3%	
Hispanic/Latinx, Black/African American	1	4.2%	
Hispanic/Latinx, Native American/ Indigenous, White	1	4.2%	
Non-Hispanic			
Non-Hispanic, White	14	58.3%	
Non-Hispanic, Asian American/Pacific Islander, White	2	8.3%	
Non-Hispanic, Black/African American, White	1	4.2%	
Non-Hispanic, Native American/ Indigenous	1	4.2%	
Education			
High school diploma or GED equivalent	4	16.7%	
Some college or associate degree	6	25%	
Bachelor’s degree	8	33.3%	
Graduate degree	6	25%	
Sexual Orientation^a^			
ACE spectrum (e.g., graysexual demisexual, asexual)	10		41.7%
Biromantic	1		4.2%
Bisexual	8		33.3%
Lesbian	6		25.0%
Pansexual	12		50.0%
Queer	11		45.8%
Same gender loving	1		4.2%
	M	*SD*	
Age (mean, *SD*)	33.0	6.5	
Range = 22.6 – 46.3			
BMI (mean, *SD*)	38.8	13.6	
Range = 18.9 – 68.8			

a Participants could select multiple responses for race and sexual orientation.

### Overview of themes

**Figure 1:**
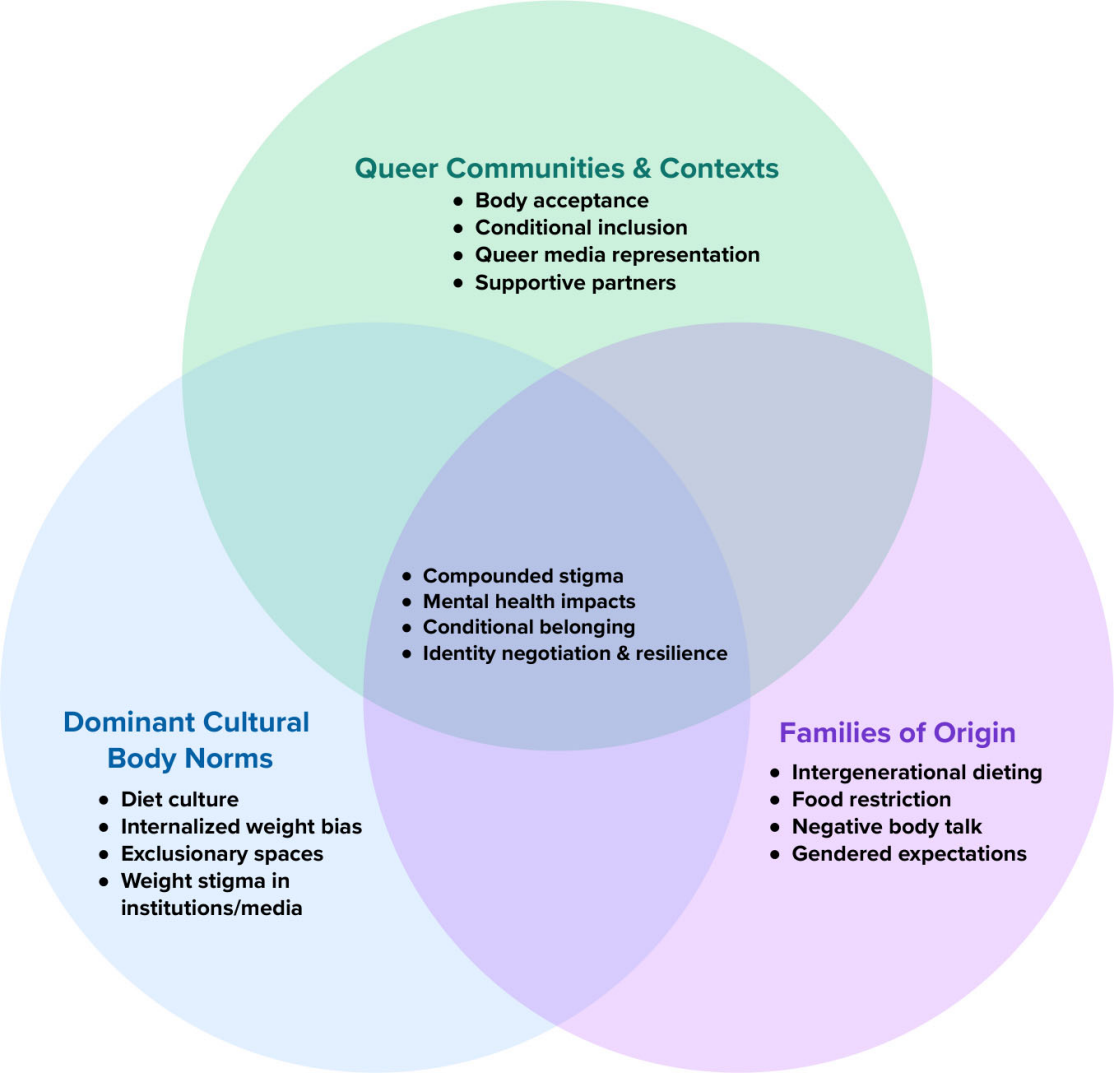
Intersecting contexts shaping body and weight-related experiences of SMW.

Participants described encountering, reinforcing, or resisting gendered body ideals across three overlapping contexts: dominant culture, families of origin, and queer communities. While each context operated in distinct ways, they often overlapped, for example, families of origin in childhood served as conduits of dominant body norms, instilling fear of fat and the moralization of body size at a young age. Participants highlighted how sociocultural norms tied women’s worth to thinness and heteronormative beauty standards, with weight stigma intersecting with gender identity, sexual orientation, disability, race, and class with cascading perceived effects on their mental health and wellbeing. For SMW in larger bodies, stigma manifested uniquely through gendered norms related to femininity, womanhood, and queer presentation. See **Figure 1** for a visual representation of the three identified themes.

### The influence of dominant cultural body norms

Participants described how dominant cultural norms shaped their understanding of bodies and weight over their life course. They routinely acknowledged that these influences contributed to their internalized anti-fat bias and diet culture (i.e., a prevailing norm equating body size with health and moralizing weight, eating, and physical activity) (34). Participants shared how these norms were encountered early in childhood, and shared through peers, media, families, health care experiences, and institutions, which taught them early on that body size was something others would monitor, praise, pathologize, exclude, or mistreat. **Table 2** provides illustrative quotes for the influence of dominant cultural body norms.

**Table 2:** Illustrative quotes of influence of dominant cultural body norms.

Weight stigma impacts on mental health	
I was bullied heavily in school. I was literally told to kill myself multiple times. … When I was like 17, 18, I started working at McDonald’s, and I just ate all the food … I hated myself so much for it. I just stopped eating. I would eat, and I would throw up…. I just felt wrong, and I stopped getting my free meals at work … [I] was so disgusted with myself.	ID 29: Lesbian, Pansexual, ACE spectrum, White/Caucasian, Non-Hispanic, 28 y/o
The older I got, the more aware of my body image and body size I became, [the] more developed those insecurities … became.	ID 40: Lesbian, Queer, Hispanic/Latinx, White/Caucasian, 30 y/o
Long-term impacts of childhood weight stigma	
So, it took me a long time to get over it. And even as I did grow up and have … intimate partners and stuff … for a long time, I felt really like, ‘Oh, I can’t believe … somebody that was so attractive would want to be with me.	ID 41: Bisexual, Pansexual, Queer, Native American/Indigenous, Hispanic/Latinx, White/Caucasian, 36 y/o
I feel like no woman would find me attractive because, like, I’m not. …[I] convinced myself, as a child, that I was basically just too fat and ugly for someone to love. And it’s bled into other parts of my life where I get too self-conscious to do the things that I actually really want.	ID 24: Pansexual, ACE spectrum, White/Caucasian, Non-Hispanic, 29 y/o
Intersectional weight stigma	
It was hard being Black, and it was hard being fat, in a town where everyone is white and not everyone is fat, especially at a young age. And I found most times that I would be more alienated for my weight than for my race, and that was hard to wrap my mind around. And of course I had race issues growing up … I was, Lord, probably seven the first time someone called me a [racial slur], so, [state in northeast region], what are you gonna do? But as I got older, my weight became more of an issue.	ID 52: Pansexual, Same Gender Loving, Queer, Black/African American, Hispanic/Latinx, 37 y/o

Participants shared how public spaces also communicated exclusionary norms. Size-inaccessible seating, transportation, and fitness environments reinforced the message that larger bodies did not belong. Participants shared that when they were growing up, the clothing options for children in larger bodies were relatively non-existent, forcing them to shop in the adult women’s section, and limiting their choice of child-appropriate, fashionable clothing styles. Participant 23 reflected, “I’d never had the clothes that the other kids had, because … they weren’t made in plus sizes. So, my clothes had to come from … the old fat lady stores … so they were very … ordinary, boring … middle-aged … church lady clothes. (ID 23: Bisexual, Queer, Black/African American, White/Caucasian, Non-Hispanic, 46 years old) Participant 20 shared how childhood experiences of interpersonal weight-related discrimination reinforced body norms, suggested that some spaces were not safe for all bodies, and discouraged participation in enjoyable, health-promoting activities:

The girls in the bleacher[s] started laughing when I took off my towel… ‘Hey, I didn’t know they allowed whales in the pool’ …I didn’t really stick around for swimming, and I didn’t go back. (ID 20: Pansexual, Queer, ACE spectrum, White/Caucasian, Non-Hispanic, 41 y/o)

Participants also discussed how early internalization of dominant narratives shaped self-perception and body image in enduring ways, leading to internalized weight bias, and damaging their mental health. For example, Participant 24 reflected on how she internalized weight bias which resulted in severe disordered eating, “I fasted and … tried to convince myself that … it was better to want to be anorexic than to just accept myself as big. I wanted to be a waif; a person who could live on six cups of coffee, a cracker or two, and nothing else.” (ID 24: Pansexual, ACE spectrum, White/Caucasian, Non-Hispanic, 29 y/o) This same participant said she “genuinely wanted that [be]cause to me, that felt like a more acceptable, better option than just being big.

Additionally, participants shared how the ramifications of weight stigma internalized during youth could continue throughout their life without resolution, consuming significant mental bandwidth through persistent intrusive thoughts. Participant 22 recounted the physical and mental toll that her relationship with food had taken on her, prompting her efforts toward eating disorder recovery:

I realized that I didn’t want to live my life in this … binge/restrict [cycle]. I started to feel a lot of digestive fallout … My relationship with food was doing violence to my body, and that’s what triggered my [eating disorder] recovery. (ID 22: Queer, ACE spectrum, Biromantic, White/Caucasian, Non-Hispanic, 47 y/o)

This participant noted that she had to find recovery through yoga philosophy due to weight stigma in eating disorder treatment and lack of affordable healthcare “both because of lack of health insurance and the size of my body, I’ve never been able to access inpatient care, even when I was probably behaviorally qualified for inpatient.” (ID 22: Queer, ACE spectrum, Biromantic, White/Caucasian, Non-Hispanic, 47 y/o)

Participants discussed how prolonged exposure to societal weight bias and intersectional body norms starting in childhood permeated different facets of their lives, ranging from their mental health to their social relationships to their sexual identity. For example, Participant 37 noted how her own internalized negative feelings about body size and poor body image affected her when she said, “[F]or a very long time, I had major depression, suicide attempts.” (ID 37: Bisexual, Pansexual, Native American/Indigenous, Non-Hispanic, 38 y/o). She also disclosed that it affected her romantic relationships throughout her life. Participants described how cultural body norms and anti-fat bias intersected with beliefs about race and disability. Participant 6, who uses a wheelchair, explained, “using mobility [devices] absolutely changes almost everybody’s perspective immediately, especially being a “not-Barbie-size” person … It’s a true stereotype… ‘You’re fat because you’re disabled … and both of those things cause each other.’” (ID 6: Queer, ACE spectrum, White/Caucasian, Non-Hispanic, 43 y/o) This intersection had impacted healthcare stigma and misdiagnosis. Participant 52 shared how racism and sizeism in healthcare impacted her life, saying.

Every health issue I’ve ever had has been contributed to my weight or to my race … I have chronic Lyme [disease] … [that] went undiagnosed for so long because the bullseye rash you get from Lyme was not visible on my skin … And it was a lot of what doctors always do to larger people—”Have you tried losing weight?”… [and] it was twofold because they didn’t know what the disease looked like on my skin tone and then … blame[d] it on my weight. It was a double whammy. (ID 52: Pansexual, Same Gender Loving, Queer, Black/African American, Hispanic/Latinx, 37 y/o)

While participants reflected on experiences of weight bias in healthcare settings leading to poor care quality, Participant 46 shared her experience facing racialized beauty standards shaping body policing: “Predominantly white people went … to my school, and a lot of the girls would critique on my body…. I wasn’t overweight. I was like a size one back then.” (ID 46: Pansexual, ACE spectrum, Hispanic/Latinx, 38 y/o). Taken together, these narratives illuminate how early, pervasive weight-centric norms shaped trajectories across health, relationships, and social systems of oppression.

### Families of origin

Messages from family members of origin and across generations emerged as particularly formative, reinforcing systemic body norms that participants internalized as anti-fat attitudes and bias. The participants described their families of origin as influential sources where weight-related messages were first learned and reinforced, shaping their understanding of bodies and weight over their life courses. These messages often validated broader societal biases observed in media, peers, and institutions (e.g., schools and healthcare). Participants shared how family members, mostly women but some men, including mothers, fathers, grandmothers, aunts, and sisters, influenced their early body image through chronic dieting, verbal comments about weight, encouragement or criticism around eating, and modeling of restrictive behaviors. This intergenerational messaging reinforced the idea that weight and body size were key measures of one’s worth. Participant 49 described the contradictory food-related messages from older generations, saying “Chinese parents or grandparents saying, ‘Why aren’t you eating more? You’re so thin,’ but then, on a dime, switching to, ‘Stop eating so much, you’re getting chubby.’” (ID 49: ACE spectrum, Lesbian, Asian/Pacific Islander, White, 26 y/o) Participant 52 shared her experience growing up in a Puerto Rican family: “The Puerto Rican side always had nicknames for those of us who were a little bit bigger, “gordita” just being “little fat girl” nickname. Hurtful, yes. Did I get over it? Probably not. But that was just kind of the talk.” (ID 52: Pansexual, Same Gender Loving, Queer, Black/African American, Hispanic/Latinx, 37 y/o). **Table 3** provides illustrative quotes for families of origin.

**Table 3:** Illustrative quotes of families of origin.

Culture & familial body messages	
[W]e’re from a Hispanic and … Native American background. … those are typically … curvier bodies. And just naturally … all my family … I did see … a larger body on them. And … I feel like, because of the relationship I had at home with my mom, it was like this … thing of … well, you don’t want to grow up to look like them … you don’t want to grow up to have that sort of shape, right? … so that definitely played a part of like … that’s … gross. That’s not right. That’s like … not pretty.	ID 41: Bisexual, Pansexual, Queer, Native American/Indigenous, Hispanic/Latinx, White/Caucasian, 36 y/o
…Chinese parents or grandparents saying, ‘Why aren’t you eating more? You’re so thin,’ but then, on a dime, switching to, ‘Stop eating so much, you’re getting chubby.’	ID 49: Lesbian, ACE spectrum, Asian/Pacific Islander, White/Caucasian, Non-Hispanic, 27 y/o
Forced restriction, weighing, and disordered eating	
She’d [participant’s mother] put me on diets. She would force me to vomit if she thought that I had too much food … I had to weigh in a lot … probably as young as five. … I don’t remember a time living with her when I wasn’t on some sort of diet.	ID 20: Pansexual, Queer, ACE spectrum, White/Caucasian, Non-Hispanic, 41 y/o
Family modeling overvaluation of appearance and weight	
All the women in my family have been highly conscious of their bodies and appearances. My mother tends to measure her life not in years, but by how much she weighed at different times.	ID 32: Lesbian, White/Caucasian, Non-Hispanic, 31 y/o
Heteronormativity	
[W]hen I was a kid … my mom … was pretty chill … but for years, she would tell me, ‘I don’t really think you’re bi[sexual] because you always … want to be with men.’	ID 15: Bisexual, Queer, Hispanic/Latinx, White/Caucasian, 34 y/o

Participants also highlighted subtle forms of sexism within their families, noting how brothers or “male presenting” siblings “didn’t have it as rough as the younger female children” (ID 42: Bisexual, ACE spectrum, Hispanic/Latinx, 23 y/o) and that [boys] didn’t have their “food restricted in the same way.” (ID 3: Pansexual, White/Caucasian, Non-Hispanic, 28 y/o). These experiences reflected how boys’ and girls’ bodies were surveilled differently, with girls often facing heightened scrutiny. Family and close others were also mentioned by participants as sources of enacted interpersonal stigma regarding body size and eating, both in childhood and adulthood. Participant 33 elaborated:

I just got more and more fat as I got older, despite the fact that I was in body conditioning for all four years of high school. I was strong, set personal records for women. I was a healthy person, but my family treated me like I wasn’t, because I was fat. And my little brother is like stick thin. We are the complete opposites, as far as body types go … Nobody ever bothered him about the amount that he would eat, but whatever I ate was always being criticized, and my dad would use terms like, ‘Go stuff your face, go eat like a pig…’ Which is just wild to think about … that adults were talking to a child that way. (ID 33: Pansexual, White/Caucasian, Non-Hispanic, 28 y/o).

This participant noted that her body size was larger or “fat” despite being young, regularly physically active, and “healthy” by her own account. She described her father’s stigmatizing language “making fun of me for my body type … calling me names”−in stark contrast to her brother’s treatment.

Participants described their families of origin as influential sources where weight-related messages were first learned and reinforced. Negative body talk was routine, particularly among women relatives: “My mom trying to … just maintain … the words that they spoke… ‘I’m so fat. I’m so fat … I ate so much. Oh, my arms are so huge’” (ID 37: Bisexual, Pansexual, Native American/Indigenous, Non-Hispanic, 38 y/o) These messages shaped their understanding of bodies and weight over their life courses, validating the negative, pervasive societal bias they observed in media, from peers, and in institutions (e.g., school, healthcare). Participant 29 shared how family experiences reinforced the violent weight-based oppression she experienced from peers saying, “Struggling with being fat, being told by my mom I was fat, her restricting food, her literally locking cabinet doors so I wouldn’t eat….” (ID 29: Lesbian, Pansexual, ACE spectrum, White/Caucasian, Non-Hispanic, 28 y/o) Participants shared experiences of the ways that diet culture was perpetuated intergenerationally, such that parents tried to monitor and control weight-related behaviors, induce weight loss for their children, and even forced them to engage in disordered eating and purging behaviors.

Family expectations around appearance often intersected with participants’ sexual orientation and gender expression. Coming out was sometimes met with conditional acceptance or dismissive comments, reinforcing feelings of scrutiny about sexuality. Participant 15 shared, “when I was a kid … my mom … was pretty chill … but for years, she would tell me, ‘I don’t really think you’re bi[sexual] because you always … want to be with men.’” (ID 15: Bisexual, Queer, Hispanic/Latinx, White, 33 y/o) Participants also discussed their emerging sexual minority identities in relation to their experiences in girlhood, and how sociocultural body-related pressures and norms had a deleterious effect on their mental health and wellbeing which persisted into adulthood. These patterns of familial messaging did not occur in a vacuum; they intersected with broader social contexts and communities that shaped how individuals experienced body image and stigma.

### Queer communities and contexts

Participants’ accounts of queer community norms around bodies suggested that they perceived affirming messages about body inclusivity and size acceptance within queer spaces. Participants’ descriptions of body-related messages in queer communities suggested that dominant cultural norms seeped into queer spaces, creating a backdrop of size-related oppression within environments they thought should be more inclusive. That is, while participants discussed the queer community in terms of its general acceptance of sexuality and gender, not all participants agreed that queer spaces were “radically inclusive space[s]” (ID 22: ACE spectrum, Queer, White/Caucasian, Non-Hispanic, 46 y/o). The participant continued: “my corner of queer community is also very accepting and accessible.” She implied that her experiences in the queer community were “unique” and accepting, diverging from typical queer spaces. Participant 40 noted that, “The queer community is more accepting, more body positive. Like, I love going to pride or to the gay clubs, and people of all shapes and sizes are wearing whatever the hell they want, and nobody’s judging them about it.” (ID 40: Lesbian, Queer, Hispanic/Latinx, White/Caucasian, 30 y/o). Supportive, inclusive queer communities were discussed in terms of fostering body acceptance and positive body image, as well as self-image. **Table 4** summarizes queer community and contexts. Participant 37 described that her experience with the queer community “seems to be so extremely accepting of everyone … it’s all inclusive and it’s all welcomed and all loved.” (ID 37; Bisexual, Pansexual; Native American/Indigenous; 37 y/o) Participant 15 shared “I think part of … what helped me feel less hatred towards myself is just some of the communities I found; the queer community, the alt[ernative] community; you know, places where your body doesn’t matter as much.” (ID 15: Bisexual, Queer, Hispanic/Latinx, White/Caucasian, 34 y/o). This participant mentioned moving away from self-hatred related to body size but could also have been reflective of other internalized bias related to their sexuality.

**Table 4:** Illustrative quotes of queer communities and contexts.

Support within queer communities	
I feel like there is a lot more acceptance of any kind of body type in the queer community.	ID 3: Pansexual, White/Caucasian, Non-Hispanic, 27 y/o
I see more openness and acceptance of my fat body in the queer, poly community than I’ve seen in any other community that I’ve ever been a part of.	ID 23: Bisexual, Queer, Black/African American, White/Caucasian, Non-Hispanic, 45 y/o
Shared queer/gender/woman experiences and body image	
I’m with someone who understands what I’m going through and understands what it’s like to be a woman and also … have issues with body image … It’s something that we’re able to really openly talk about. And it feels a lot less isolating [be]cause I’m not comparing in the way of, like, ‘Oh, I wish I looked like you,’ but more so, like, we’re comparing our experiences and sharing our experiences and being able to support each other through that. And I think that’s honestly, like, really, really helpful, because it doesn’t feel so shameful.	ID 32: Lesbian, White/Caucasian, Non-Hispanic, 31 y/o
Queer media and representation	
I think … something that I enjoy about the queer community … is that I do see a very wide variety of, like, body shapes represented in social media…	ID 47: Bisexual, Queer, Asian/Pacific Islander, White/Caucasian, Non-Hispanic, 30 y/o
…In my books that I write, my characters, my girl heroines are always bigger because I do want to be a part of that representation in media.	ID 15: Bisexual, Queer, Hispanic/Latinx, White/Caucasian, 34 y/o
Cisnormativity, heteronormativity, gender norms and weight stigma	
If you’re large [and queer] then you’re supposed to be … butch or non-binary, but because I like to be feminine and large, that’s a no, and people wouldn’t like that.”	ID 24: Pansexual, ACE spectrum, White/Caucasian, Non-Hispanic, 29 y/o

Queer relationships and partners also were places where support was found, and a shared female identity seemed to allow for a deeper connection around body image issues, a possible protective factor against weight stigma among some queer women with women/femme/queer partners. One participant explained how her partner supports her:

And every time I … brought it up sometimes, like, ‘Oh, I look fat in this dress, Oh, I look like this’, she [participant’s partner] would always tell me, ‘No, you look beautiful, I don’t see what you see.’ So that made me feel a little bit better about myself because she never talked down to me about my body … I think the other aspect of it is that someone from my culture doesn’t think I’m fat, accepts me for who I am, and is nurturing and caring … it fulfilled that little me inside that lingered for someone to say something like that, that was a female. (ID 46: Pansexual, ACE spectrum, Hispanic/Latinx, 38 y/o).

Participant 46’s use of the word “fat” with a negative connotation conflicts with other participants’ uses of the word “fat” as a neutral descriptor of body size, demonstrating internalized weight bias.

Participants’ descriptions of body-related messages in queer communities suggested that dominant cultural body norms seeped into queer spaces, and intersected with gender presentation, race, and disability to create unique community experiences across social categories. Participant 40 explained how she felt her gender expression might be protective against weight stigma, stating, “For me, personally, I have not experienced a lot of … appearance-based discrimination related to being queer. And I think that has a lot to do with being more femme presenting.” (ID 40: Lesbian, Queer, Hispanic/Latinx, White/Caucasian, 30 y/o) Participant 41 observed how the Pride event in her area in the Western U.S. was not accessible for disabled or chronically ill people who have significant mobility issues: “[it] is so not accessible … there [are] steps everywhere.” (ID 41: Bisexual, Pansexual, Queer, Native American/Indigenous, Hispanic/Latinx, White/Caucasian, 36 y/o) Participant 52 discussed the challenges she encounters related to representation and discrimination at intersections of sizeism, racism, and homophobia:

Gayness in the Black community is difficult … I feel like being white and gay is something so celebrated and so welcomed … if you are fat and Black and gay, you have to be the self-appointed punchline to be accepted, and if you’re not, you are an outcast, or you are not fun to be around. If you are not the life of the party, if you are not the one who drinks too much, if you don’t have a designated spot as a fat, Black, queer person, you’re kind of left to the side, especially with outside communities like the white community. I mean they want someone who’s like, “Oh my God, I want my own Billy Porter.”…If you’re not entertaining, you are not valid as a larger Black person. (ID 52: Pansexual, Same Gender Loving, Queer, Black/African American, Hispanic/Latinx, 37 y/o)

Together, participants highlighted the ways that queer communities upheld broader systems of oppression, which intersected with weight stigma to create unique and compounded experiences of exclusion and harm.

Participants also observed how queer media spaces upheld appearance and body size ideals for SMW, demonstrating how dominant cultural body norms intersected with queer body norms and weight stigma, stunting body acceptance in these spaces. Participant 20 shared, “Your worth is how skinny you are … even in a lot of the … lesbian media … there was a really big emphasis on how you looked.” (ID 20: Pansexual, Queer, ACE spectrum, White/Caucasian, Non-Hispanic, 41 y/o) Participants also described how queer media and digital content perpetuated community-specific ideals of bodies, beauty, and health, where even body-positive content often centered white, non-disabled, conventionally attractive, mid-size bodies, rather than larger plus-size bodies less represented in mainstream fashion. Participant 33 noted that there is an “ideal lesbian woman that is heavily perpetuated by things like TikTok, who is thin, with long hair, and she’s masc[uline] but not too masc, because then it’s too manly … it becomes a sort of inherent biphobia and fatphobia, and they’re tied together, because they’re both impacted by social media.” (ID 33: Pansexual, White/Caucasian, Non-Hispanic, 27 y/o) This reflects concerns about media-enforced standards perpetuating weight-based oppression and bi-erasure.

Participants also talked about the ways that coming to understand and accept their sexuality and queer identity helped them nurture more positive body image:

[O]nce I acknowledged that I was queer, my goal was always to be … happy with myself … That also comes in the realm of body positivity, [be]cause if I wanted to feel comfortable with my queer identity, I also wanted to feel comfortable with my body and also be kind to myself … Those kinda tied in together … If I can accept my queerness, I can accept my body as it is.” (ID 42: Bisexual, ACE spectrum, Hispanic/Latinx, 23 y/o)

Navigating homophobia along with body-related oppression allowed participants to draw parallels between stigmas, recognizing how stigma, whether related to sexual identity or body size, harmed their self-image and well-being. As Participant 40 explained: “Realizing that I was a lesbian also made a huge impact on my body image. [Bec]ause I was like, I love all bodies, why wouldn’t they love mine?” (ID 40: Lesbian, Queer, Hispanic/Latinx, White/Caucasian, 30 y/o) She noted how accepting her queerness was intertwined with unlearning internalized anti-fat bias, and that finding self-kindness involved understanding and accepting all aspects of herself. She continued, “[A]lso realizing that the queer gaze is far, far different than the straight cis male gaze,” (ID 40: Lesbian, Queer, Hispanic/Latinx, White/Caucasian, 30 y/o), suggesting that navigating queer attraction fostered greater body acceptance. Participant 49 (Lesbian, ACE spectrum, Asian/Pacific Islander, White/Caucasian, Non-Hispanic, 27 y/o) describes how queer spaces feel protective for her body image and freeing from dominant cultural body norms:

[Being queer] has been freeing … If I were … trying to … meet … heterosexual beauty standards … I would probably have worse body image than I do now … It’s nice to know that one, my girlfriend finds me attractive kind of no matter what I look like, and two, even if we weren’t dating, I can … dress however I want, and you know there’d be a lesbian out there who was into it.

These narratives collectively illuminate how queer contexts can both challenge and reproduce body norms, underscoring the complexity of body image within diverse queer communities.

## Discussion

In this study, we examined how this sample of cisgender SMW experience weight stigma and sociocultural body norms using semi-structured interviews and an intersectional, weight-inclusive framework and life history approach. Participants’ accounts illustrated how weight stigma was embedded within cultural, familial, and queer community contexts, operating as a pervasive and at times violent force that reinforced size, gender, race, and class hierarchies. While some queer spaces fostered greater body acceptance and challenged heteronormative beauty ideals, others reproduced size-based exclusion and oppression, underscoring how cisgender SMW navigate complex and at times contradictory sociocultural messages about bodies that both reinforce and resist weight stigma.

This study is unique in its departure from mainstream public health discourse on SMW and weight in that we center weight *stigma* as a public health problem that is *exacerbated* by conventional public health research, which typically pathologizes SMW, as well as racially minoritized women, who have higher BMIs. Scholarship on weight stigma is frequently embedded within weight-centric approaches that focus on single-axis experiences of interpersonal or internalized bias and overlook how weight stigma intersects with heterosexism, racism, ableism, and classism (35). By drawing on intersectional, life course, and minority stress approaches to analyze and interpret participants’ accounts, the present study shifts our attention away from individual weight control and towards the structural and cultural systems that shape body image and health. This alternative focus highlights unique stigma configurations and shared mechanisms of exclusion that can shape population-level variability in health and enhance public health strategies for population-based health (e.g., culturally sensitive weight-inclusive anti-bullying campaigns).

In considering health promotion for SMW, larger-bodied participants mentioned that disordered eating behaviors were encouraged and at times forced on them as children by their parents and doctors. Participants reflected on these experiences as traumatic and harmful, with mental health impacts lasting into adulthood. These early life experiences reflect how minority stress processes may extend beyond heterosexism to include weight stigma, compounding distal stressors such as interpersonal discrimination from families with proximal ones (e.g., internalized homophobia and weight bias). These findings align with research documenting greater body dissatisfaction and eating concerns among sexual minorities, especially gay men and bisexual women, compared to heterosexual peers (36, 37).

The recent findings have important implications for prevention and clinical care. These narratives underscore a need for routine, stigma-informed screening practices in clinical and school settings for early detection of and intervention for disordered eating symptomology and its sequelae (38). In the context of eating disorder care, participant narratives reinforce the need for addressing weight bias not as a single-axis issue but one that is compounded by sexual orientation, gender, race, ability, and class. For individuals with eating disorders, using affirming, weight-inclusive, patient-centered approaches that are informed by anti-racist, intersectional understandings of body image can address treatment inequities (39). While our analysis emphasizes structural stigma, individual differences in emotion regulation and self-conscious emotions (e.g., guilt/shame) may shape how weight stigma is internalized and experienced (40, 41).

Participants’ accounts demonstrated that queer spaces were not immune to size-based exclusion. Many cisgender SMW described how body size intersected with gender presentation and race to shape belonging, where some larger-bodied feminine-presenting women described feeling subject to heighted scrutiny, while others encountered assumptions that queer women in larger bodies should adopt more masculine or androgynous styles. This illustrates how queer spaces may present an intersectional paradox such that they may resist dominant gender and sexuality norms, while simultaneously upholding or reproducing sizeism and racialized beauty standards. Minority stress theory may help explain how intra-community exclusions could create cumulative and compounding stressors and conditions that mitigate the protective potential of queer community. Research with sexual minority communities, and particularly SMW, has found mixed evidence about the protective nature of queer communities for body image (42–44). Findings from this study echo previous mixed findings (45), demonstrating both affirming and exclusionary dynamics within queer spaces, relationships, and communities. These findings highlight the need to consider weight stigma not only as a product of dominant cultural ideals, but also as a process embedded within queer communities and subcultures that intersects with broader systems of oppression (e.g., sexism, racism, heterosexism). Mixed findings regarding the protective nature of queer community and queer identity may be reflective of this; where communities that tend to challenge traditional gender presentation norms may be relatively more inclusive but still exist within a larger system of intersectional weight-based oppression.

These findings point to actionable implications for health promotion among SMW, and the population more broadly, by targeting modifiable structural determinants. For example, public health promotion should give greater attention to structural ways of supporting inclusive norms, such as advocating for queer-affirming children’s clothing that is available in diverse sizes. Public health promotion should also involve greater advocacy for policy-level interventions that protect children and adults from weight-based discrimination. These strategies align with both minority stress and intersectionality frameworks by shifting the focus from individual behaviors to structural-level factors.

Educational programs for queer youth could address body diversity alongside sexuality and gender, interrupting sizeist norms before they are reproduced within community spaces. Incorporating weight inclusivity into public health and school-based anti-bullying campaigns could extend current allyship approaches that address sexual orientation and gender identity toward intersectional, inclusive curricula. Healthcare and clinical practice settings should ensure that spaces are affirming for all identities as well as body sizes, and DEI training should include weight-inclusive education. Addressing weight stigma structurally can disrupt the pathways through which intersecting forms of oppression (e.g., heterosexism, ableism, racism) lead to poor health and well-being.

Weight stigma researchers should place the emphasis on modifiable social-structural factors that perpetuate the interlocking oppressive systems that harm people in larger bodies and contribute to health inequities rather than locating the issue of weight stigma within an individual. Intersectionality researcher Lisa Bowleg (2023) reminds us that, “the remedy for racialized health inequities is not to change the “race” of people oppressed by structural racism, it is to dismantle structural racism” (p. 106) (46). The mental health harms of weight stigma have been evidenced (47), yet still “non-stigmatizing” weight management and lifestyle intervention is upheld as a way to mitigate the impacts of weight stigma, while still viewing “fixing” large people’s bodies (however “kindly”) as the goal. The current findings demonstrate how greater attention toward the effects of interlocking social-structural inequality on health could refocus the intervention priority away from a problem of the individual[‘s body] to the totality of ways in which our society fosters body size-related discrimination through mutually reinforcing inequitable systems (e.g., healthcare, media, education, employment, access to public spaces) that in turn reinforce discriminatory beliefs, values, and distribution of resources (48). Participants in this study discussed the ways that health-promoting spaces (e.g., gyms, swimming pools) were inaccessible for and/or exclusionary of larger bodies, creating oppressive barriers to life-enhancing movement. For example, one participant’s record-setting body was viewed as less worthy than her thin brother’s body, leading to a relationship with her body marked by shame and surveillance. These accounts illustrate how weight stigma operates as a distal minority stress, where structural exclusion and interpersonal discrimination fosters internalized stigma (a proximal minority stressor). From an intersectional perspective, these experiences do not exist in isolation, but are compounded by gendered and familial expectations and messaging that position larger-bodied SMW differently from their peers. Addressing these inequities requires structural changes that communicate acceptance of larger bodies and challenges sizeist norms (e.g., by advocating for availability of athletic clothes in larger sizes and reframing physical activity spaces to emphasize inclusion rather than weight loss).

Our study is not without limitations. The thematic framework in the present study is limited by author positionality including that the first author and many co-authors use a weight-inclusive, body liberation lens. The sample was adult cisgender women, majority (58%) Non-Hispanic White, U.S.-based, and middle-aged. The results may not be transferable to other populations, including transgender and non-binary individuals, who may face distinct forms of stigma, particularly as it relates to cisnormativity within body ideals. While intersectionality played a role within the results, particularly with respect to sexual orientation, further work explicitly examining additional intersectional forces of discrimination (e.g., race, disability, etc.) would be needed to determine how those facets of identity and resulting privilege/oppression shape experiences of weight stigma. Additionally, many life-course qualitative approaches rely on longer and repeated interviews; with additional time we could have elicited more depth from participants, particularly when exploring how weight stigma impacted older individuals in the later decades of their lives.

Greater knowledge of these factors, particularly for at-risk groups like SMW, can offer insight into broader mechanisms influencing mental health risk and enhance public health strategies for population-based health. By centering the lived experiences of SMW in this discourse, this study demonstrates how weight stigma operates intersectionally, intertwined with gender, sexual orientation, class, and ability. Integrating these insights with minority stress theory extends the framework beyond sexuality to show how multiply marginalized populations face compounded forms of stress and oppression impacting health inequities, highlighting intervention targets relevant to many different axes of oppression.

## Data Availability

The raw data supporting the conclusions of this article will be made available by the authors, without undue reservation.
