# Supplementary material for: “If I can accept my queerness, I can accept my body as it is”: Understanding weight-related perspectives and stigma from sexual minority women
Source: Front Psychiatry. 2025 Oct 2;16:1687680. doi: 10.3389/fpsyt.2025.1687680 (PMC12528178; doi:10.3389/fpsyt.2025.1687680)
Supplement: Supplementary file 1 [file Table1.docx]

**Appendix A. Semi-Structured Interview Guide**

1. I want to take a moment to learn more about your identities and communities that are most important to you. It could include things like race, ethnicity, gender, sexual orientation, social class, abilities, health conditions, faith or religion, rurality or location, body size, or family role. Could you share a few of these now?

**Body-related Messages - Past**

1. I'd like to talk about the different ways that you might have experienced or noticed how bodies and weight were talked about or communicated about growing up. Can you tell me about a memorable time when you were made aware of your body or someone else’s and it being different?
2. Growing up, did you feel that there were certain expectations for how body shapes or sizes should be for people?
3. What messages did you receive from your family about bodies (your own or others), weight management, and/or dieting growing up?
   1. How, if at all, do you think these experiences or the messages your family conveyed were related to [previously disclosed social identity or race/ethnicity, if applicable] you or they hold?
   2. How, if at all, do you think these experiences or the messages your family conveyed were related to gender or sexual identities that you or they hold?
4. What kind of messages about weight, size, or bodies did you hear from your friends and/or social groups?
5. In what ways do you think the media (including social media, if applicable) you were exposed to (such as movies, television, magazines) contributed to your understandings of weight and body size?

**Body-related Messages - Present**

1. In what ways, if at all, do you feel like those messages and the way you experienced them growing up showed up for you as you got older?
2. What kind of messages about weight, size, or body do you hear from your friends and/or social groups? What about specifically from the LGBTQ+ community?

**Weight-related Stigma and Intersections**

1. Have you personally experienced, noticed, or been told about any kinds of weight-related discrimination?
   1. Have you experienced or noticed this kind of body- or size-related prejudice or discrimination within the queer community?
   2. How if at all do you think these experiences of body-related discrimination or prejudice are related to your gender identity or gender expression?
   3. Have you experienced or noticed body-related discrimination that looks or feels different because of your or someone’s sexual identity or queer presentation?
   4. Have you noticed or experienced body-related discrimination or prejudice that feels related to your or someone’s race or ethnicity?
   5. Have you noticed or experienced any other kinds of discrimination or prejudice that you attributed to your or someone else’s body shape or size that might also be related to a marginalized identity that you or they hold? This might be related to ableism, healthism, classism, racism, sexism, transphobia, etc.

**Wrap-Up**

1. Are there any other things you can think of that have contributed to your understanding of bodies and weight that we haven’t touched on yet or is there anything else you would like to share?
